# Scaling Effects on Single‐Cell Manipulation Using Magnetic Forces at Edge of Flat Plate With Elliptically Micro‐Projection

**DOI:** 10.1002/bit.70057

**Published:** 2025-09-02

**Authors:** Satoshi Ota, Hiroki Yasuga, Takeshi Akagawa, Yuta Kurashina, Kenta Nakazawa, Shoichi Kikuchi

**Affiliations:** ^1^ Department of Electrical and Electronic Engineering Shizuoka University Hamamatsu Japan; ^2^ Self‐Care Device Research Team (SDRT), Integrated Research Center for Self‐Care Technology (IRC‐SCT), National Institute of Advanced Industrial Science and Technology (AIST) Ibaraki Japan; ^3^ Electrical and Electronic Engineering Course, Graduate School of Integrated Science and Technology Shizuoka University Hamamatsu Japan; ^4^ Division of Advanced Mechanical Systems Engineering, Institute of Engineering Tokyo University of Agriculture and Technology Tokyo Japan; ^5^ Department of Mechanical Engineering Shizuoka University Hamamatsu Japan

**Keywords:** magnetic force, magnetic nanoparticles, microfabricated flat plate, single‐cell manipulation, surface tension

## Abstract

Single‐cell manipulation is needed for various cellular analyses and biomedical applications. In the conventional single‐cell manipulation methodology, cells are the center of a flat dish or hollow space, where the medium spontaneously penetrates. However, manipulation at the edge of the plate, where the flow of the medium is interrupted by its surface tension, has not yet been shown. In this study, we manipulated single cells labeled with magnetic nanoparticles at the edge of a projecting microfabricated plate under a magnetic field, which offer a less invasive method for cellular analyses, not limited by the shape of the culture devices. As a cell is affected by both the surface tension of the medium and magnetic force, we investigated the effects of the shape and scale of the projection on cell motion. Owing to energy minimization between the liquid–air and solid–liquid interfaces determined by the structure of the projection, the area of solution spreading in the projection converged, and we obtained design guidelines for the projection that enabled cell manipulation. Demonstrating accuracy in single‐cell manipulation on a microfabricated flat plate with different projection shapes contributes to developing single‐cell analyses, including applications such as single‐cell‐based polymerase chain reactions.

## Graphical abstract

To develop the single‐cell manipulation on the flat plate, cells labeled with magnetic nanoparticles were manipulated using magnetic force for less invasive cellular analyses at the edge of a projecting microfabricated plate. The optimal shape and scale of the projection was investigated considering the effects of the flow in the droplet on the plate and magnetic field distribution.

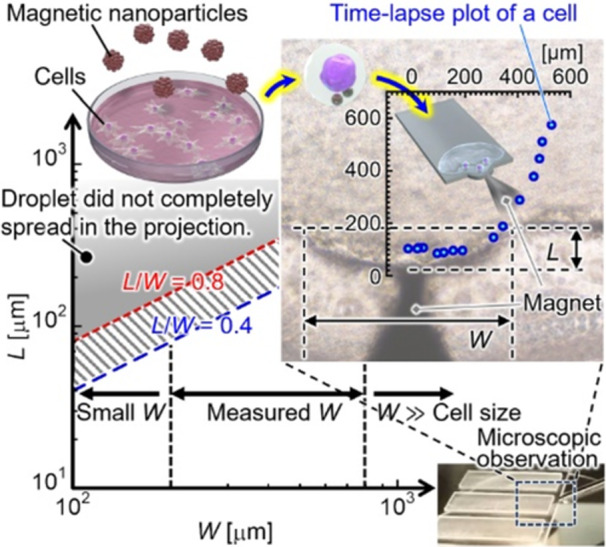

## 1 Introduction

Single‐cell manipulation and analysis are necessary for evaluating cellular functions in biomedical applications. Owing to the unique biology of individual cells, single‐cell western blotting analysis (Kang et al. 2016) and single‐cell RNA sequencing (Hwang et al. 2018) have been investigated. In particular, cell manipulations on micro‐substrates that are fabricated based on the process of microelectromechanical systems (MEMS) are attracting attention (Yi et al. 2006; Huang et al. 2014). The various techniques of single‐cell manipulation on the micro‐substrates include robotic micromanipulation (Zhou et al. 2004; Solano and Wood 2007; Kim et al. 2012) and noncontact micromanipulation based on optical, (Ashkin et al. 1987; Chiou et al. 2005; Hayakawa et al. 2016) electrical, (Voldman 2006; Park et al. 2010; Huang et al. 2016) acoustic, (Ding et al. 2012; Ahmed et al. 2016; Imashiro et al. 2016; Imashiro et al. 2018) fluidic, (Lee et al. 2004; Yan et al. 2004; Wheeler et al. 2003) and magnetic forces (Yamamura et al. 2005; Carlo et al. 2006; Ino et al. 2008; Hagiwara et al. 2010; Betal et al. 2018). Robotic manipulation using a MEMS actuator results in poor biocompatibility, causing damage to proteins around cells due to contact with the MEMS actuator, for example, a micro‐gripper in biological fluid. Regarding manipulation using optical and electrical forces, the intensity of laser and electric field irradiation, respectively, is limited by thermal damage to cells. In contrast, although acoustic and fluidic manipulations are relatively less invasive, manipulation is influenced by acoustic wave mode and the shape of the culture device. In the fluidic system, the influence of the shape of the device is limited because the cells are trapped on fabricated platforms.

In cell manipulation using magnetic force, cells are labeled with magnetic nanoparticles (MNPs), which have also attracted attention as carriers in drug delivery systems, magnetic separation, and heat sources for cancer hyperthermia treatment (Pankhurst et al. 2009; Krishnan 2010). A static magnetic field is applied using an electromagnet or permanent magnet such as an Nd‐Fe‐B magnet. Although an electromagnet can flexibly control a magnetic field, its field strength is limited to a smaller value than that of a permanent magnet. Ito et al. proposed a tissue engineering methodology using magnetically labeled cells and magnetic force as magnetic force‐based tissue engineering (Ito et al. 2005). For cell patterning using a magnetized thick steel plate (Ino et al. 2007) and fabricated magnet array (Ino et al. 2008), cells were rapidly cultured in a line or curve, respectively, and formed arrays of single adherent cells. Yan et al. showed the methodology for manipulating a single DNA molecule using a small permanent magnet as a magnetic tweezer (Yan et al. 2004). Betal et al. demonstrated targeted cell manipulation using MNPs as nanorobots, remotely controlled by a magnetic field (Betal et al. 2018). Moreover, direct manipulation of intracellular structure in single cells was conducted using MNPs three‐dimensionally controlled by a magnetic field inside a cell (Wang et al. 2019). Micromanipulation based on magnetic force was relatively less invasive and was not limited by the shape of the culture device. In addition to individual cell manipulation, magnetic labeling of living cells creates opportunities for numerous biomedical applications, such as tracking by magnetic resonance imaging (Wilhelm and Gazeau 2008) and magnetic particle imaging (Zheng et al. 2016). The tumor microenvironment was characterized by from the magnetic relaxation time of MNPs dependent on the viscosity in surroundings (Ota et al. 2024). Thus, the methodology for manipulating magnetically labeled cells can be integrated with other biomedical techniques. Besides, the label‐free microfluidic cell manipulation in the magnetic medium through negative magnetophoresis was also investigated (Zhao et al. 2016).

In the conventional methodology, cells are guided and manipulated into the center of a flat dish or hollow space, where the medium spontaneously penetrates. Manipulation at the edge of the plate, where the flow of the medium is interrupted by its surface tension, has not been shown. When droplet‐enclosed cells are added onto a microfabricated plate, the increase in surface tension or viscosity affects culture medium penetration and hinders cell manipulation. Moreover, although a single cell can be manipulated close to the liquid–air interface, it is difficult to place and immobilize the cell at the point of interest.

In this study, we demonstrate cell manipulation on microfabricated plates with semi‐ellipsoidal projections using MNPs and a magnetic field. The effects of scaling on cell culture, cell proliferation, and cell assembly characteristics were investigated using microfabricated substrates (Kikuchi et al. 2014; Mizutani et al. 2014). The effects of the shape and scale of the plate projection on the accuracy of single‐cell manipulation investigated herein contribute to the framework of cell manipulation using magnetic force, for example, the design of microfabricated plates, the amount of MNPs, and the required magnetic field gradient.

## 2 Materials and Methods

### 2.1 Study Design

**Figure 1:**
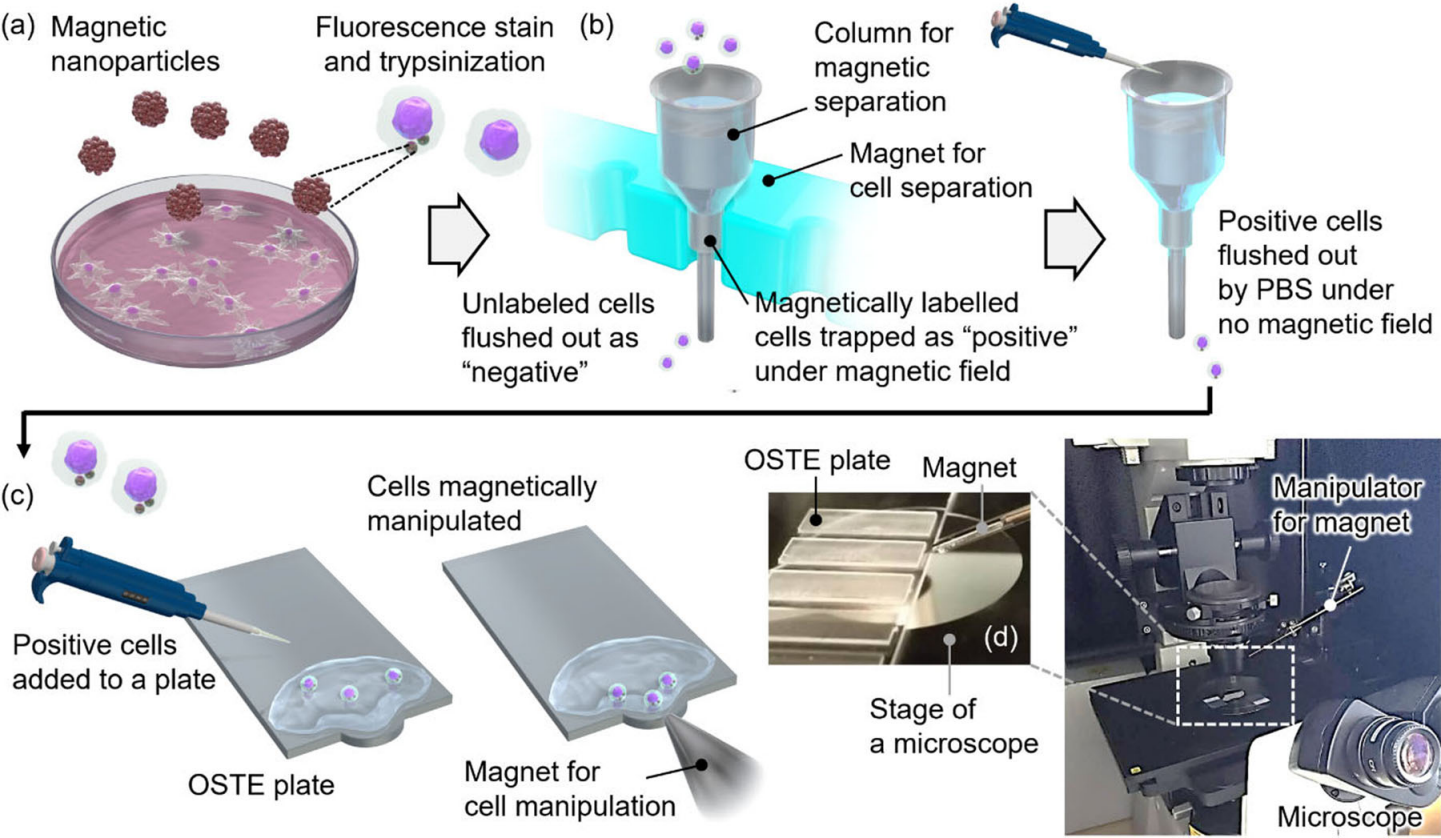
Schematics of (a) cell culture with magnetic nanoparticles, (b) magnetic separation of cells labeled with magnetic nanoparticles, and (c) cell manipulation system on a microfabricated plate using a permanent magnet. (d) Photo of the microfabricated off‐stoichiometric thiol–ene (OSTE) plates placed on the stage of the microscope and the permanent magnet approaching the edge of a plate using the manipulator installed on the microscope.

Figure 1 shows the schematics of the cell culture and manipulation system using MNPs on a microfabricated plate under a magnetic field. The MNPs were internalized into cultured cells for magnetic labeling (Figure 1a). After the fluorescent labeling and trypsinization of the cells, they were magnetically separated (Figure 1b). Magnetically positive cells were added onto the plate and manipulated using a magnet (Figure 1c). The manipulation was conducted on the stage of an inverted microscope under a fluorescence microscope (Figure 1d). The plate was fabricated using a photocurable polymer system, off‐stoichiometric thiol–ene (OSTE), wherein fluorescent cells were observed using an inverted microscope because of the transparency and low autofluorescence of the OSTE plate (Yasuga et al. 2021). The experimental details are presented in the following sections.

### 2.2 Preparation of Microfabricated Plates

**Figure 2:**
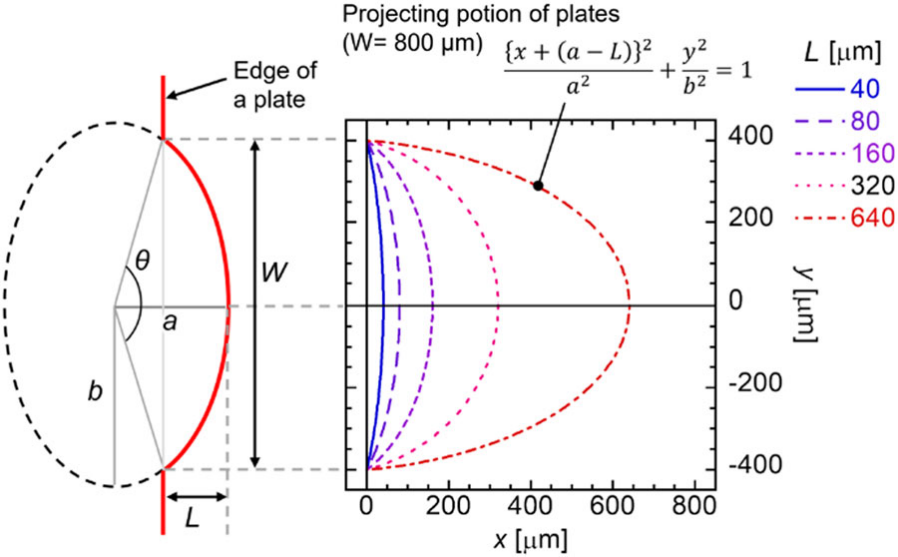
Schematic depicting the design strategy of the projection of the plate. The curve for the projection was estimated using Equation (2), which represents an ellipse. Two kinds of radii, *a* and *b*, of the ellipse were calculated based on the width *W* and length *L* of the projection and the angle of the arc in the ellipse, that is, *θ* = 148°. The graph shows the example of the projection design at *W* = 800 μm.

Figure 2 shows the design strategy of the plate edge where the projection was fabricated. The shape of the projection, scaled at width *W* and length *L*, was determined based on the curve of the ellipse, calculated as follows:

(1) $$\frac{{\left\{x+\left(a-L\right)\right\}}^{2}}{a^{2}}+\frac{y^{2}}{b^{2}}=1,$$

where *a* and *b* represent the radius of the ellipse in *x* and *y* axes, respectively, calculated based on *W* and *L* listed in Table 1 with the fixed angle of the arc in ellipse, *θ* = 148°, and the origin of the plotted curves (Figure 2) was set at the base of the projection along the *x* axis and the center of the ellipse. The elliptic shape of the projection was derived from the curve shaped by the droplet including cells added on the plate. It is necessary to sufficiently penetrate the droplet along with the edge of the projection, which is explained in Section 3.2 in detail.

**Table 1:** List of the width *W* and length *L* of the projection and the ratio *W*/*L* for the fabricated plates named P1–13. The plates marked with the asterisk symbol (*) were used in single‐cell manipulation experiments.

	*W* [μm]	*L* [μm]	*L*/*W*	
P1	200	20	0.1	*
P2	200	40	0.2	*
P3	200	80	0.4	
P4	200	160	0.8	
P5	400	40	0.1	*
P6	400	80	0.2	*
P7	400	160	0.4	
P8	400	320	0.8	
P9	800	40	0.05	*
P10	800	80	0.1	*
P11	800	160	0.2	*
P12	800	320	0.4	
P13	800	640	0.8	

OSTE, a photocurable polymer system, was selected as the plate material for cell manipulation. The use of OSTE is advantageous in this study because of its excellent characteristics of high resolution, transparency, and biocompatibility that help in fabrication (Carlborg et al. 2011). We used OSTEMER 322 Crystal Clear resin (Mercene Labs AB, Stockholm, Sweden) as an OSTE material. The steps involved in the fabrication of the OSTE plate are presented below. First, we prepared a photomask that included the plate patterns under the conditions summarized in Table 1 (Takeda Tokyo Process Service, Kanagawa, Japan). Next, the OSTE polymer precursor was poured onto a flat glass substrate (Matsunami Glass Ind. Ltd., Osaka, Japan). Approximately 450 µm‐thick glass spacer was placed around the OSTE precursor, and a photomask was placed onto the spacer to secure the space with 450 µm height. The OSTE precursor was exposed to collimated ultraviolet (UV) for 30 s. After UV exposure, the formed plate structures were immersed and developed in isobutyl acetate via ultrasonication. Finally, the substrates were removed and dried at room temperature.

In previous studies, the hydrophilicity of the same OSTE materials by measuring the water contact angle was examined, which reported to vary from 67° to 99° (Aubrecht et al. 2024; Sandström et al. 2015; Zhou et al. 2017). This range of mean values likely reflects differences in fabrication conditions. In contrast to the considerable differences in mean contact angles, the coefficient of variation (CV) to be below 5% was reported. Since the same OSTE materials was used in these studies, we expect that our fabricated plates will exhibit a similarly low CV.

### 2.3 Surface Coating and Measurement of MNPs

γ‐Fe_2_O_3_ nanoparticles (primary diameter = 29 nm) were purchased from CIK NanoTek Co. Tokyo, Japan. These nanoparticles were coated with polyethyleneimine max (PEI) with a molecular weight of 40,000 (Polysciences Inc. Warrington, PA). Nanoparticles (200 mg) were dispersed in a 10 mL solution of 1.0 mg/mL PEI via ultrasonication for 10 min. This solution was purified by centrifugation at 1000 × *g* for 15 min. The supernatant was centrifuged at 10,000 × *g* for 30 min, and the precipitate was collected as PEI‐coated MNPs, which were positively charged in diluted water (Ota et al. 2013). The structure of the PEI‐coated MNPs and their coating layer were observed using a transmission electron microscope (TEM) at the Hanaichi UltraStructure Research Institute (Okazaki, Japan) in Supporting Information S1: Figure S1.

The average hydrodynamic diameter of the PEI‐coated MNPs was 76.9 nm, and their polydispersity index was 0.22 (Supporting Information S1: Figure S2), which was measured by dynamic light scattering using a fiber optical particle analyzer (ELSZ­2000ZS; Otsuka Electronics Co. Ltd., Osaka, Japan). The biocompatibility of PEI‐coated MNPs was confirmed by measuring cell viability which was no longer decreased by adding PEI‐coated MNPs as shown in Supporting Information S1: Figure S3. PEI‐coated MNPs were used as carriers of magnetofection, which were internalized into cells via endocytosis (Ota et al. 2015a).

The magnetization of these MNPs as a function of the applied magnetic field strength was measured using a vibrating sample magnetometer (VSM) at 0–1.6 T. The basic system of the VSM, including an electromagnet, sample vibrator, and pickup coils, was manufactured by Riken Denshi Co., Tokyo, Japan. Current was supplied to the electromagnet using a bipolar power supply (Kudo Electric Co. Ltd., Sendai, Japan), controlled by a function generator (WF1983; NF Corporation, Yokohama, Japan). The strength of the applied magnetic field was measured using a teslameter (F41; Lake Shore Cryotronics Inc. Ohio, USA). The magnetization signal detected by the pickup coils was measured using a lock‐in amplifier (LI5645; NF Corporation, Japan) and recorded using a Memory HiCorder unit (MR6000‐01) and a digital volt meter (MR8990) purchased from Hioki EE Corporation, Ueda, Japan. PEI‐coated MNPs were fixed using epoxy resin at the concentration of 1 mg/mL in a cylindrical holder of 70 μL because the physical motion of MNPs was almost inhibited in the cytoplasm and on the cell membrane (Ota et al. 2015b).

### 2.4 Use of a Permanent Magnet for Cell Manipulation

**Figure 3:**
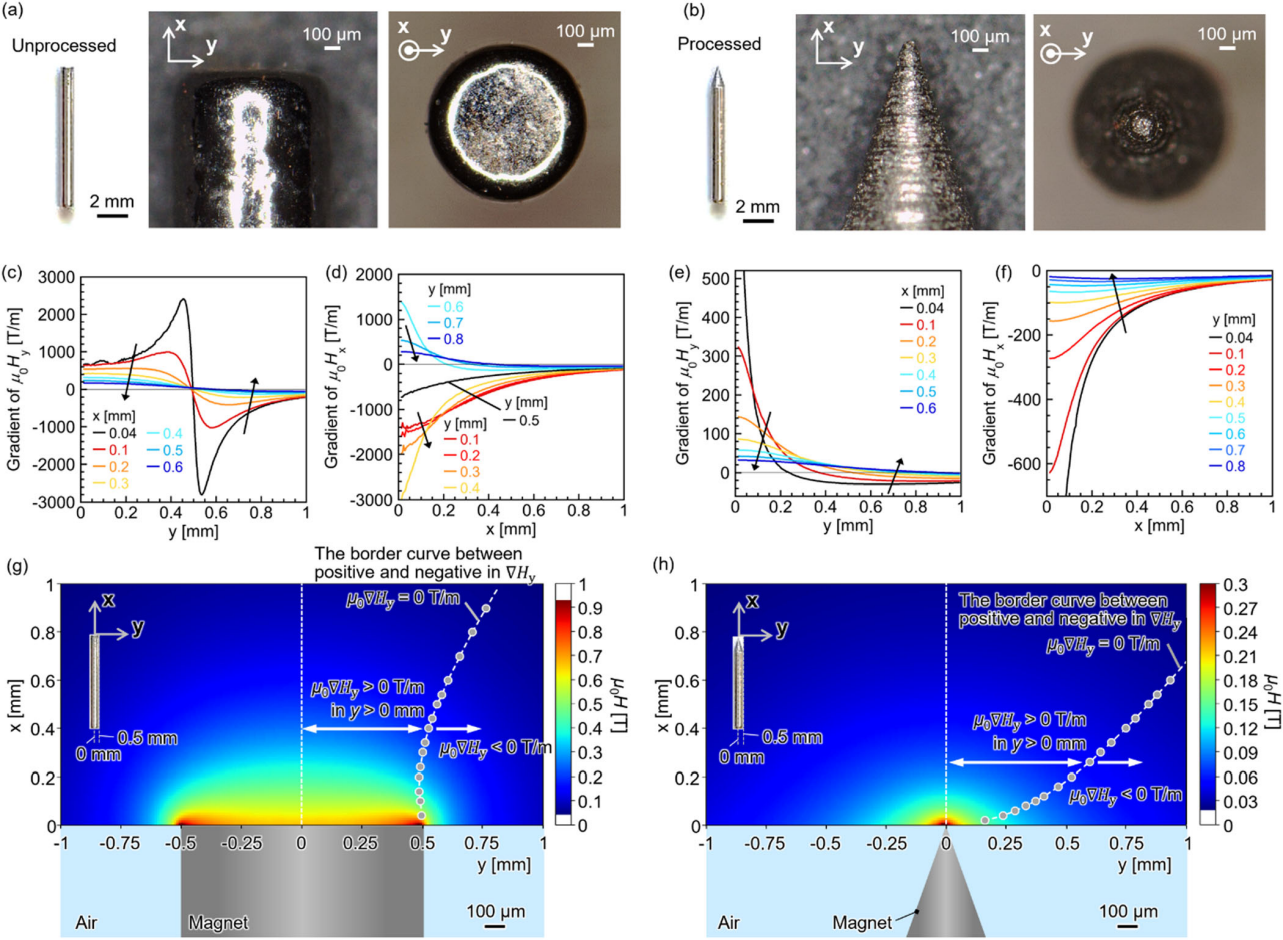
Schematics and optical micrographs of the magnified point images of (a) unprocessed and (b) processed Nd–Fe–B permanent magnets. The simulated gradient of magnetic flux density *μ*_0_ *H*, *μ*_0_ ∇ *H*, on the (c) *y*‐axis at each *x*‐position and (d) *x*‐axis at each *y*‐position in the unprocessed Nd–Fe–B permanent magnet, and *μ*_0_ ∇ *H* on the (e) *y*‐axis at each *x*‐position and (f) *x*‐axis at each *y*‐position in the processed Nd–Fe–B permanent magnet. The simulated *μ*_0_ *H* on an *xy*‐plane in the (g) unprocessed and (h) processed Nd–Fe–B permanent magnets. The dotted curves with solid circles indicate the position where the *μ*_0_ ∇ *H* on the *y*‐axis, *μ*_0_ ∇ *H*_y_, was zero as the border between positive and negative in ∇ *H*_y_, in *y* > 0 mm. The distribution of *μ*_0_ *H* was symmetrical about *y* = 0 mm.

Figure 3a shows optical micrographs of the Nd–Fe–B permanent magnets purchased from Sangyo Supply Co., Sendai, Japan, and processed into a needle shape by KRI Inc. Kyoto, Japan as shown in Figure 3b. The diameter of each magnet was set as 1 mm. The peak angle of the acute magnet was 38°. In cell manipulation experiments, the processed magnet was used considering the direction of the motion of cells estimated based on finite‐element simulation performed using JMAG Designer Ver. 24 (JSOL Corporation, Tokyo, Japan).

### 2.5 Cell Manipulation Experiments on Microfabricated Plates

Mesenchymal stem cells were cultured in minimum essential medium Eagle‐alpha modification supplemented with 10% fetal bovine serum and 1% penicillin–streptomycin in a humidified‐atmosphere incubator at 5% CO_2_ and 37°C. The cells were seeded in 35‐mm dishes at a density of 100,000 cells per 1 mL of medium on the day before the magnetic separation process. Next, MNPs mixed with 1 mL of culture medium were added to the cells at a concentration of 2 μg/mL and incubated for 24 h, following which the medium was removed, and the cells were washed with phosphate‐buffered saline (PBS). Subsequently, the cells were fluorescently labeled using a 1.0 mM solution of acetomethoxyl ester of calcein in dimethyl sulfoxide (Dojindo Molecular Technologies Inc., Kumamoto, Japan) and detached by trypsinization in 0.050% trypsin–EDTA. When an excessive amount of MNPs was added to the cells, the MNPs aggregated in the culture medium and were absorbed onto the cell surface, physically hindering the cell manipulation process. It has previously been reported that approximately 2 μg of PEI‐coated MNPs are effectively internalized in cells (Ota et al. 2013).

The detached cells in solution were loaded onto an MS column (Miltenyi Biotec KK, Tokyo, Japan) for magnetic‐activated cell sorting under a magnetic field (Kami et al. 2014). The column was washed using PBS to remove the cells wherein the MNPs were not internalized and absorbed onto the cell surface. The cells retained in the column under the magnetic field were flushed out with the culture medium using a plunger supplied with the column without a magnetic field. The flushed cells contained MNPs and were considered MNP‐positive.

The microfabricated plates were placed on the stage of an inverted microscope (Eclipse Ti; Nikon Solutions Co. Ltd., Tokyo, Japan). For cell manipulation, after the permanent magnet was placed around the micromanipulator (M‐152; Narishige, Tokyo, Japan) installed on the microscope, 10 μL of solutions containing 10 or 100 cells were dropped onto the plate under fluorescent observation.

## 3 Results and Discussion

### 3.1 Effects of the Processing of Permanent Magnets

The magnetic force *F*_*M*_ [N] was calculated as follows:

(2) $$F_{M}=\mu_{0}H\mathrm{MV}\nabla H,$$

where *M* [*A/mA*/*m*] is the magnetization of MNPs, *V* [m^3^] is the volume of MNPs, *μ*_0_ *H* [*T*] is magnetic flux density, and *μ*_0_ ∇H [T/m] implies a gradient of *μ*_0_ *H*, which indicates that magnetically labeled cells are moved by *F*_M_ in a gradient magnetic field. *μ*_0_ = 4*π* × 10^−^^7 ^H/m is the permeability in free space.

Figure 3c and d shows ∇ *H* in *y‐* and *x*‐axes, that is, ∇ *H*_y_ and ∇ *H*_x_, dependent on the *y*‐ and *x*‐distances from the center of the unprocessed magnet, respectively. The magnets were magnetized along the *x*‐axis. In *μ*_0_ ∇ *H*_x_ and *μ*_0_ ∇ *H*_y_ < 0 T/m, magnetically labeled cells were guided toward the center of the magnet. ∇ *H*_y_ was changed the values from positive to negative, and ∇ *H*_x_ was changed the values from negative to positive when close to and distant from the centers of the magnets with the boundary around *y* = 0.5 mm of the edge of the unprocessed magnet in the *y*‐axis, respectively.

In the processed magnet, ∇ *H*_y_ was changed the values from positive to negative when close to and distant from the centers of the magnets (Figure 3e). ∇ *H*_x_ was negative in 0 mm < *y* ≤ 0.8 mm, as its strength exhibited a monotonic decrease with increasing distance from the magnet particularly in *y* ≤ 0.4 mm (Figure 3f).

Figure 3g,h shows *μ*_0_ *H* distributed on an *xy*‐plane in unprocessed and processed magnets, respectively. Toward the edge of the magnets, magnetic flux was concentrated as shown by locally increased *μ*_0_ *H*, which is also illustrated by increasing the intensity of ∇ *H*_y_ and ∇ *H*_x_ shown in Figure 3c–f. The cells were displaced along the position on the *y* axis where ∇ *H*_y_ was zero, and the polarization of ∇ *H*_y_ was changed as shown by the plots in Figure 3g,h. While *μ*_0_ *H* was reduced after processing because of the decrease in the volume of the magnet, the cells were guided at the single position determined by the single edge of the magnet by the processing. It is difficult to guide the cells at the single position by the unprocessed magnet because of its concentric edge. It is important for the accurate cell manipulation to concentrate magnetic flux at the single position smaller than the size of a cell.

In the conventional research, the core material of electromagnets (Matthews et al. 2004; Wang et al. 2020) and ferritic stainless steel magnetized using permanent magnets (Stevens et al. 2024) were processed into needle shapes to improve accuracy of the cell manipulation. An electromagnet needle was also used as the actuator for magnetic droplet manipulation (Yang et al. 2018).

The processed magnet was set at the center of the plate projection along the *y*‐axis. Notably, ∇ *H*_y_ was positive and negative inside and outside the plate, bordering on zero ∇ *H*_y_. When cells located at zero ∇ *H*_y_ were attracted by negative ∇ *H*_x_ toward the negative direction in the *x*‐axis, they were affected by negative *F*_M_ and were reattracted to the position at zero ∇ *H*_y_. Thus, when cells were affected by *F*_M_ alone, they moved on to the curve at zero ∇ *H*_y_.

### 3.2 Limit of Cell Approaching Point on the Flat Projecting Plate

**Figure 4:**
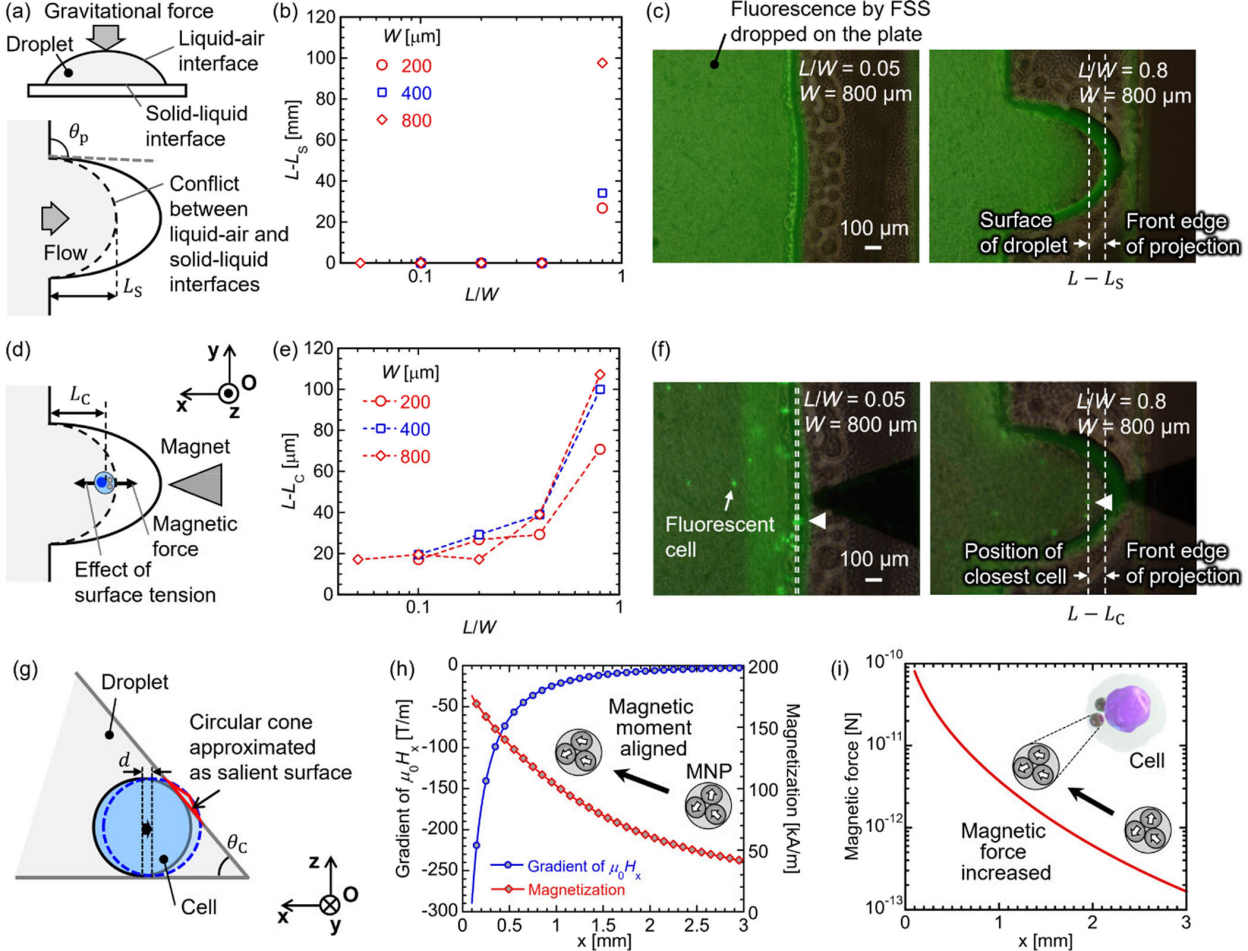
(a) Schematic depicting the droplet on the plate and the flow of solution on the projection. (b) Dependence of the distance between the surface of the droplet and the base of the projection, *L* – *L*_S_, on the *L*/*W* ratio in the experiment using fluorescein sodium salt (FSS). (c) Phase contrast images of the plate overlapped with fluorescent images of FSS dropped onto the plate at the *L*/*W* ratio = 0.05 and 0.8 at *W* = 800 μm. (d) Schematics of a cell affected by magnetic force and the effect of surface tension of the droplet on the plate. (e) Dependence of the distance between a cell closest to the front edge of the projection and the base of the projection, *L* – *L*_C_, on the *L*/*W* ratio when 100 cells were added. (f) Phase contrast images of the plate overlapped with fluorescent images of cells attracted to the projection. White arrowheads indicate the cell closest to the edge of the projection. (g) Image of a magnetically labeled cell at the edge of the droplet pulled out by magnetic force. (h) Dependence of the gradient of the applied magnetic field calculated by the finite‐element simulation and initial magnetization curve of polyethyleneimine‐coated magnetic nanoparticles (MNPs) measured by the vibrating sample magnetometer, on the distance from the edge of the permanent magnet. (i) Dependence of the magnetic force calculated using Equation (2) on the distance from the edge of the permanent magnet.

First, the curve shaped by the droplet was observed under the fluorescence of the fluorescein sodium salt (FSS) solution. The droplet model is shown in Figure 4a. When the dropped solution was spread onto the plate under gravitational force and surface tension, a sessile drop flowing into the projection was formed, and the shape of the sessile drop converged to a certain form due to energy minimization between the liquid–air and solid‐liquid interfaces in the projection. Figure 4b shows the peak of the droplet surface on all plates listed in Table 1 when 10.0 μL of FSS solution was dropped onto the plates. The droplet was approached at the edge of the plate at an *L*/*W* ratio ≤ 0.4 regardless of each value of *L* and *W*. The fluorescent image at *L*/*W* = 0.8 illustrates that the solution did not completely spread in the projection (Figure 4c). Moreover, at *L*/*W* = 0.8, the distances up to which the droplet spread from the base of the projection, that is, *L*_S_, were 133, 286, and 542 μm (the ratio *L*_S_/*L* = 0.67, 0.71, and 0.68) at *W* = 200, 400, and 800 μm, respectively. The similar *L*_S_/*L* ratios at each *W* were associated with the effects of the solid–liquid interface determined by the angle of the base of the projection, *θ*_p_, which was similar at the same *L*/*W* ratios.

On a flat plate, cells were dispersed and enclosed within a droplet of PBS, where the movement of the cells was restricted by surface tension (Figure 4d). As shown in Figure 4e, the position of the cell closest to the front edge of the projection was measured in an excess of 100 cells added to the plate under a magnetic field. The limitation of cell approaching point in the droplet was evaluated ignoring stochastic effects of disturbing the position of cells by including enough cells in the droplet. The distance between a cell and the base of the projection, that is, *L* – *L*_C_, increased with increasing *L*/*W* ratios at *W* = 200 and 400 μm, whereas the values of *L* – *L*_C_ at *L*/*W* = 0.05, 0.1 and 0.2 with *W* = 800 μm were similar. It is assumed that the number of cells approaching the front edge of the projection was influenced by not only *L*/*W* ratios but also *W*. Cells approaching the droplet surface under magnetic force were pushed back because of surface tension. As the magnetic force applied to the cell was not sufficiently large to deform the droplet, the cells were enclosed within the droplet.

### 3.3 Effects of Surface Tension and Magnetic Force at the Edge of the Droplet

The surface tension of the droplet on the flat plate was calculated as follows: (Takahashi and Masumoto 2021)

(3) $$\gamma =\rho g\left(\frac{h_{D}}{{\tilde{h}}_{D}}\right),$$

where *ρ* is the density of the solution, *g* = 9.8 m/s^2^ is the acceleration of gravity, *h*_D_ is the height of the observation point from the top of the droplet, and ${\tilde{h}}_{D}$ = 2sin(*θ*/2) is the nondimensional height of the droplet. Moreover, *θ* is the angle between the tangential line at the observation point on the surface of the droplet and the base line of the droplet parallel to the *x*‐axis. When a cell moves on the plate surface, *h*_D_ equals the height of the droplet at 0.968 mm and *θ* = *θ*_C_ = 50°, the contact angle between the basal plane of the droplet and the plate (Figure 4g), as the diameter of a cell is considerably smaller than *h*_D_, where *γ* = 12.9 mN/m in *ρ* = 1004 kg/m^3^ of PBS. The contact angle was measured in the photograph of the PBS droplet on the OSTE plate (Supporting Information S1: Figure S4). Here, the energy derived from surface tension required for the increment in the area of the droplet surface was calculated as *E*_S_ = 60.9 fN·m when a cell with a diameter of 20 μm was pulled out using magnetic force at the distance *d* = 1 nm, thoroughly smaller than the size of the cell itself. The locally salient surface of the droplet was approximated as a circular cone to simplify geometric considerations, as shown in Figure 4g.

The energy, *E*_M_, generated by the magnetic force in the *x*‐axis was estimated using Equation (2) for *d* = 1 nm. Figure 4h shows the dependence of ∇ *H*_x_ calculated based on the finite‐element simulation and *M* measured using VSM on *x*‐distance. The coercivity of the MNPs, measured based on the full magnetization curve, was 3.08 mT, sufficiently smaller than *H*_x_ within the diameter of the droplet, indicating that magnetic moments reversed within the droplet (Supporting Information S1: Figure S5). The magnetization at 1.6 T was 251 kA/m, close to the saturated value.

Here, when 2 μg of MNPs were added to 240,000 cells, 8.3 pg of MNPs were available to a cell. The *F*_M_ estimated using Eq. (2) synergetically increased with decreasing the distance from the front edge of the magnet under the effects of enhancement of ∇ *H*_x_ and *M* caused by increasing *H*_x_ (Figure 4i). When the cell was pulled out using magnetic force at *d* = 1 nm in *x* = 100 μm, *E*_M_ = 80.8 aN·m, which was approximately 7500 times smaller than *E*_S_, which also indicates that the magnetic force was overly small for the deformation of droplet surface. To increase the magnetic force for the penetration of the droplet surface, it is necessary to impractically increase the amount of MNPs added to the cell and both the strength and gradient of the applied magnetic field in this experimental setup.

### 3.4 Statistical Analysis for Single‐Cell Manipulation Experiments

For single‐cell manipulation, 10 cells were placed on a plate under a magnetic field. After the droplet including cells was added on the plate, the manipulated cells were observed, and the droplet was removed to clean the plate for the next test repetitively conducted. As the likelihood of a cell approaching the front edge of the projection was affected by stochastic factors, for example, the first position of the cells when dropped on the plate and the disturbed flow in the droplet, the position of the manipulated cell was evaluated by repetitive tests. A repetitive test was conducted using the plate marked with the asterisk symbol in Table 1 to exclude the case wherein the motion of the cell was interrupted by the surface tension of the droplet, where the solution completely spread on the projection. The distance between the cell and the front edge of the projection was evaluated using the lengths *L*_SC_ whose components in *x‐* and *y‐*axes were *L*_x_, and *L*_y_.

**Figure 5:**
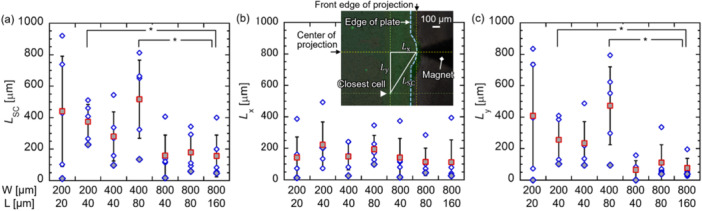
(a) *L*_SC_, (b) *L*_x_, and (c) *L*_y_ in the plates P1, P2, P5, P6, P9, P10, and P11 in Table 1. *L*_SC_ is the distance between the front edge of the projection and the closest cell. *L*_x_ and *L*_y_ are the components of *L*_SC_ in *x‐* and *y‐*axes. The diamond shape indicates the position of a cell in the five cases of comparatively small *L*_SC_ in the repetitive test. Solid diamonds indicate cases with the smallest *L*_SC_. Solid squares indicate the averaged value in the repetitive test (*p < 0.05). The inset shows the phase contrast images of the plate overlapped with fluorescent images of cells dropped onto the plate at *W* = 400 μm and *H* = 80 μm for evaluation in single‐cell manipulation.

Figure 5b–d shows *L*_SC_, *L*_x_, and *L*_y_, respectively, tested five times when a cell was comparatively close to the front edge of the projection in the trials more than 10 times. Compared with the smallest *L*_SC_ for each plate, *L*_SC_ took the smallest value for the smallest *L*/*W* ratio when *W* was the same. It is assumed that disturbance of the flow in the droplet on the projection was reduced at small *L*/*W* ratios. The averaged *L*_SC_ in each plate took a small value at *W* = 800 μm.

The value of *L*_SC_ at *W* = 800 μm was significantly smaller than that of *L*_SC_ at *W* = 200 and 400 μm for the same *L*/*W* ratio = 0.2 when estimated by Welch's *t*‐test (*p* < 0.05). With small *L*/*W* ratios and large *W* values, it was relatively simple for a cell to approach the targeting position, indicating that a quasi‐large *L* was required for stable cell positioning. In addition, a retention effect of the flow induced by the pressure gradient toward the projection was expected. The trend of *L*_SC_ was similar to that of *L*_y_, whereas the *L*_x_ values for all plates were similar. It is indicated that it was difficult to place a cell at the center of the *y‐*axis, as it was pushed out of the area around the center of the *y‐*axis where ∇ *H*_y_ > 0 as shown in Figure 3e.

### 3.5 Discussion for Accurate Single‐Cell Manipulation on Flat Plate

**Figure 6:**
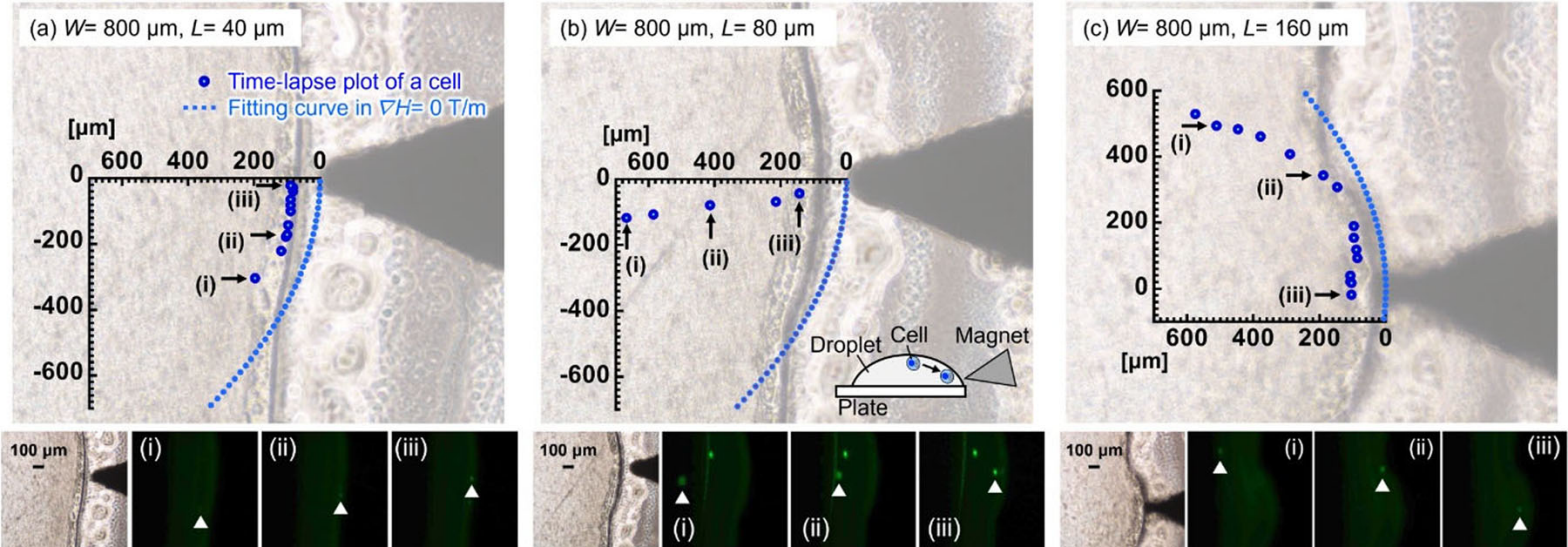
Time‐lapse plots displaying the position of a cell and the corresponding fluorescent images on the plate with *W* = 800 μm and *L* = 40 μm (a), 80 μm (b), and 160 μm (c) when a cell significantly approached the front edge of the projection. The plots displaying every (a, b) 2 s and (c) 4 s from when a fluorescent cell was visually observed by fluorescent microscopy.

Figure 6 shows the time‐lapse plots and images of the position of a cell on a plate of *W* = 800 μm when a cell sufficiently approached the front edge of the projection. The curve illustrates the border between zero ∇ *H*_y_. At *L* = 40 and 160 μm, a cell was traced at the edge of the projection owing to *F*_M_ directed in the *x*‐axis by ∇ *H*_x_ and the hydrodynamic force *F*_H_ pulling cells in the projection such as the capillary force due to the surface tension compressing the droplet around the projection (Figure 6a,c) Notably, *F*_H_ applied to a cell around the projection was larger than *F*_M_, as the cell moved against the direction of *F*_M_ in the *y‐*axis associated with ∇ *H*_y_.

As shown in Supporting Information S1: Figure S6, in the large *W* = 800 μm, cells were pulled into the projection at the short *L*_y_. By contrast, in the small *W* = 200 and 400 μm, cells were guided to the position of distant *L*_y_ by the large *F*_M_ associated with ∇ *H*_y_, which is illustrated by *L*_y_ > *L*_x_ in zero ∇ *H*_y_. Cells gradually sank and were pinned on the surface of the plate in the position of the distant *L*_y_. In Figure 6c, the cell was disturbed by an unsteady flow around the front edge of the projection.

At *L* = 80 μm, a cell was traced around the center of the *y*‐axis. A moving cell came into focus in the fluorescent images when it approached the front edge of the projection (Figure 6b), indicating that this cell was placed in a direction perpendicular to the plate. The cell moved along the curve based on the shape of the droplet.

**Figure 7:**
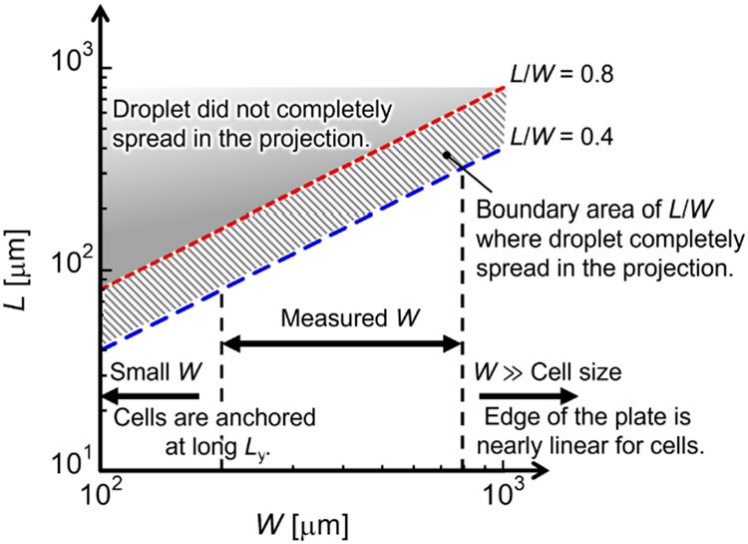
Phase diagram displaying the effects of varying *L* and *W* values on single‐cell manipulation.

Figure 7 shows the effects of varying *L* and *W* values on single‐cell manipulation. At *L*/*W* ratio > 0.8, the cell did not approach the edge of the projection because the droplet did not completely spread on the projection. When *W* was small, the cell was anchored at a long *L*_y_, as shown in Figure 5d. Although the *L*_x_ values were similar regardless of *L* and *W*, the difference in *L*_SC_ was significantly influenced by *L*_y_ (Figure 5). In contrast, when the *W* of the projection was significantly greater when compared with the size of a cell, the edge of the plate was nearly linear for the cell, which reduced the accuracy of single‐cell manipulation because a cell was anchored at a long *L*_y_ as was the case for small *W*.

In a particular case wherein a cell was not accurately manipulated at the front edge of the projection, the cell unsteadily moved upward and flowed off under the strong effect of the Marangoni flow at the liquid–air interface (Giorgiutti‐Dauphiné and Pauchard 2018) and went out of focus, blurring the image (Supporting Information S1: Figure S7). It is assumed that the temperature at the surface of the droplet decreased due to heat loss by the evaporation. At that time, the temperature at the surface closer to the edge, that is, the contact line, remained relatively higher due to heat absorption from the plate, while that at the apex decreased more strongly because of the larger path lengths from the plate. The negative surface tension gradient in the direction from the apex to the edge, which was caused by the positive temperature gradient, induced a positive Marangoni number as shown in Figure S7b (Supporting information) (Hu and Larson 2005). The Marangoni number is defined as the dimensionless number that illustrates the intensity and direction of the Marangoni flow. The temperature gradient increased as the contact angle increased when the hydrostatic pressure associated with gravity was negligible compared to the effects of surface tension for a small droplet (Hu and Larson 2005; Girard et al. 2006). It is also indicated that the Marangoni convection occurred due to the positive Marangoni number for the droplet contact angle of 50° substantially larger than 14° where the sign of the Marangoni number was changed (Hu and Larson 2005). Temperature variation is one of the possible factors that affect cell manipulation. The employment of a high‐humidity chamber covering experimental setups would reduce the rate of evaporation, contributing to the construction of a more robust system.

In addition to following off under the effect of the Marangoni flow, when a cell settles at the front edge of the projection, it occasionally flows off under the effect of the viscous flow caused by another cell approaching it. It is shown that a cell can flow off under the effect of the Marangoni flow beyond pinning under the magnetic force.

Cells were manipulated on the flat plate using magnetic force in a low‐invasive manner, avoiding direct physical contact with manipulation tools. One limitation of the study is the challenge of securely retaining a cell at the edge of the plate, as cells are free from physical stress and susceptible to internal flow within the droplet. It is therefore necessary to optimize both the material and projection shape of the plate, taking into account the influence of the flow acting on a cell.

## 4 Conclusion

We demonstrate that the accuracy of single‐cell manipulation depends on the shape of the microfabricated projection at the edge of a flat plate. The ratio of the length *L* and width *W* of the projection determined the extent to which the solution dropped onto the plate spread into the projection because of energy minimization between the liquid–air and solid–liquid interfaces in the projection. Cells attracted by the magnetic force were enclosed within a droplet of the solution, wherein the cells were dispersed. Conversely, magnetically labeled cells could not penetrate the droplet surface, as theoretical estimation indicates that the energy generated by the magnetic force is significantly smaller than that required for penetration by overcoming surface tension. Theoretical estimation also indicates that the penetration of a cell into the droplet surface is difficult in an actual magnetic field gradient and the amount of MNPs internalized into the cytoplasm.

In the single‐cell manipulation experiment, the number of cells approaching the front edge of the projection when the droplet completely spread to the front edge of the projection was affected by *W*. Considering droplet spreading, a cell is likely to approach a position close to the front edge of the projection at small *L*/*W* ratios and large *W*. Moreover, around the front edge of the projection, the disturbance in the motion of a cell indicates that the hydrodynamic force dominantly affects the motion of a cell rather than the magnetic force.

The effects of the shape and scale of the plate projection on the accuracy of single‐cell manipulation investigated herein contribute to the framework of cell manipulation using magnetic force, for example, the design of microfabricated plates, the amount of MNPs, and the required magnetic field gradient. The demonstration of single‐cell manipulation on a projecting microfabricated plate, where the penetration of the medium is inhibited, is crucial for developing accurate single‐cell analyses, such as single‐cell‐based polymerase chain reaction. In future studies, it will be necessary to consider the material of the microfabricated plate that affects the quality of droplet spreading on the plate, such as the wettability and roughness of the surface of the plate.

## Author Contributions

**Satoshi Ota:** conceptualization, methodology, formal analysis, investigation, resources, data curation, writing – original draft preparation, visualization, project administration, funding acquisition. **Hiroki Yasuga:** conceptualization, methodology, investigation, resources, writing – original draft preparation, writing – review and editing. **Takeshi Akagawa:** methodology, investigation. **Yuta Kurashina:** conceptualization, methodology, investigation, writing – review and editing, visualization, funding acquisition. **Kenta Nakazawa:** conceptualization, methodology, investigation, writing – review and editing. **Shoichi Kikuchi:** conceptualization, methodology, investigation, writing – review and editing, supervision, funding acquisition.

## Conflicts of Interest

The authors declare no conflicts of interest.

## Supporting information


**Figure S1:** TEM images of (a) PEI‐coated MNPs and (b) their coating layer negatively stained. **Figure S2:** Hydrodynamic diameter of PEI‐coated MNPs measured by a DLS. **Figure S3**: Viability of HeLa cells in adding PEI‐coated MNPs. HeLa cells were seeded in 35‐mm dishes at a density of 100,000 cells/well. **Figure S4:** Photograph of the PBS droplet on the OSTE plate. *θ*_C_ is the contact angle between the basal plane of the droplet and the plate. *h*_D_ is the height of the droplet. **Figure S5:** Magnetization curve of PEI‐coated MNPs solidified by epoxy resin under a direct current magnetic field. Inset shows the magnified graph around the original point. **Figure S6:** (a) Distribution of the magnetic field strength *μ*_0_ *H* in a cell position from the edge of the projection in *x*‐ and *y*‐axis, *L*_x_ and *L*_y_, respectively. **Figure S7:** (a) Phase contrast images of the plate overlapped with fluorescent images of a cell manipulated on the plate at *W*=800 μm and *H*=160 μm.

## Data Availability

The authors have nothing to report.
