# Supplementary material for: Scaling Effects on Single‐Cell Manipulation Using Magnetic Forces at Edge of Flat Plate With Elliptically Micro‐Projection
Source: Biotechnol Bioeng. 2025 Sep 2;122(12):3355–65. doi: 10.1002/bit.70057 (PMC12599492; doi:10.1002/bit.70057)
Supplement: Supplementary file 1 — Figure S1: TEM images of (a) PEI‐coated MNPs and (b) their coating layer negatively stained. Figure S2: Hydrodynamic diameter of PEI‐coated MNPs measured by a DLS. Figure S3: Viability of HeLa cells in adding PEI‐coated MNPs. HeLa cells were seeded in 35‐mm dishes at a density of 100,000 cells/well. Figure S4: Photograph of the PBS droplet on the OSTE plate. θ C is the contact angle between the basal plane of the droplet and the plate. h D is the height of the droplet. Figure S5: Magnetization curve of PEI‐coated MNPs solidified by epoxy resin under a direct current magnetic field. Inset shows the magnified graph around the original point. Figure S6: (a) Distribution of the magnetic field strength μ 0 H in a cell position from the edge of the projection in x‐ and y‐axis, L x and L y, respectively. Figure S7: (a) Phase contrast images of the plate overlapped with fluorescent images of a cell manipulated on the plate at W=800 μm and H=160 μm. [file BIT-122-3355-s001.docx]

Supporting Information

Scaling Effects on Single-Cell Manipulation Using Magnetic Forces at edge of Flat Plate with Elliptically Micro-Projection

Satoshi Ota^*^^,†^, Hiroki Yasuga^†^, Takeshi Akagawa, Yuta Kurashina, Kenta Nakazawa, and Shoichi Kikuchi

^*^ Corresponding author: Satoshi Ota; E-mail: ota.s@shizuoka.ac.jp

^†^First author: Satoshi Ota and Hiroki Yasuga


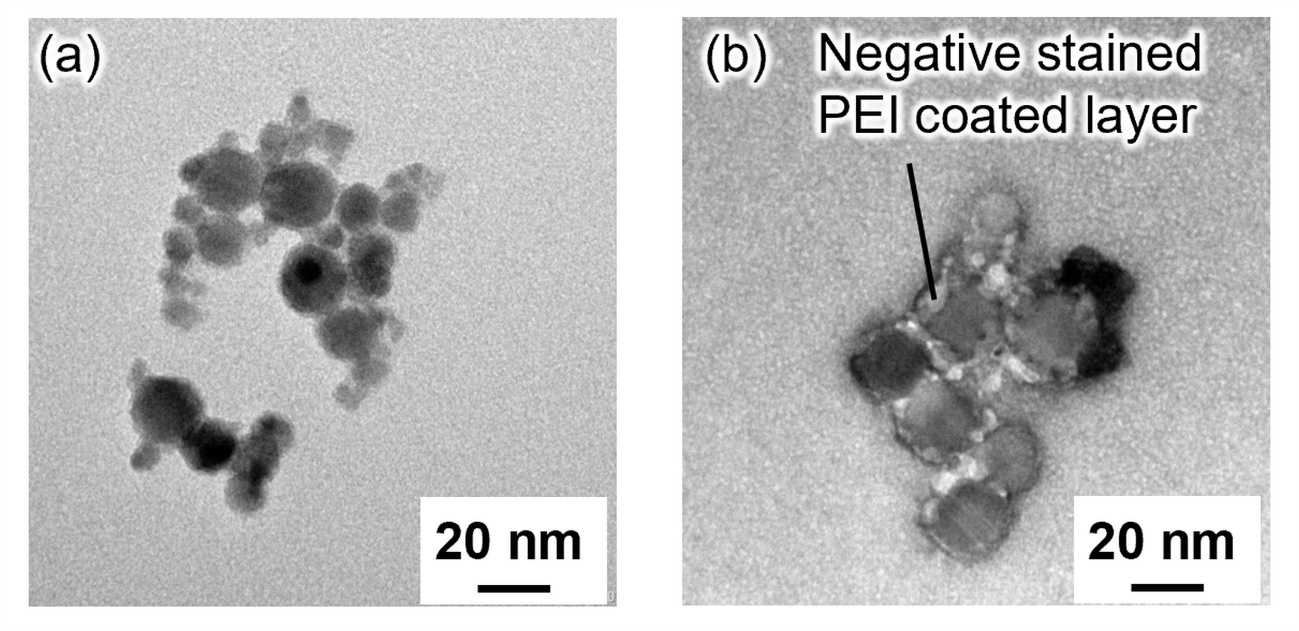


**Figure S1.** TEM images of (a) PEI-coated MNPs and (b) their coating layer negatively stained.


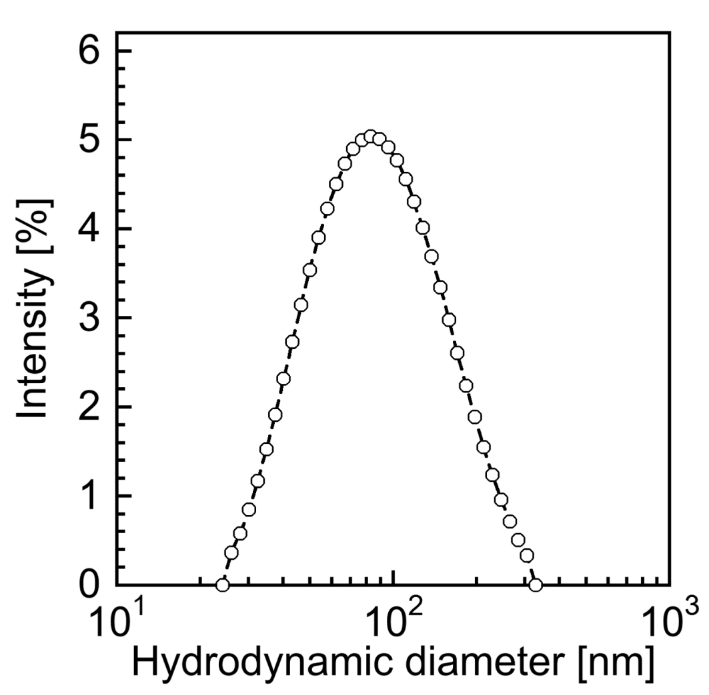


**Figure S2.** Hydrodynamic diameter of PEI-coated MNPs measured by a DLS.


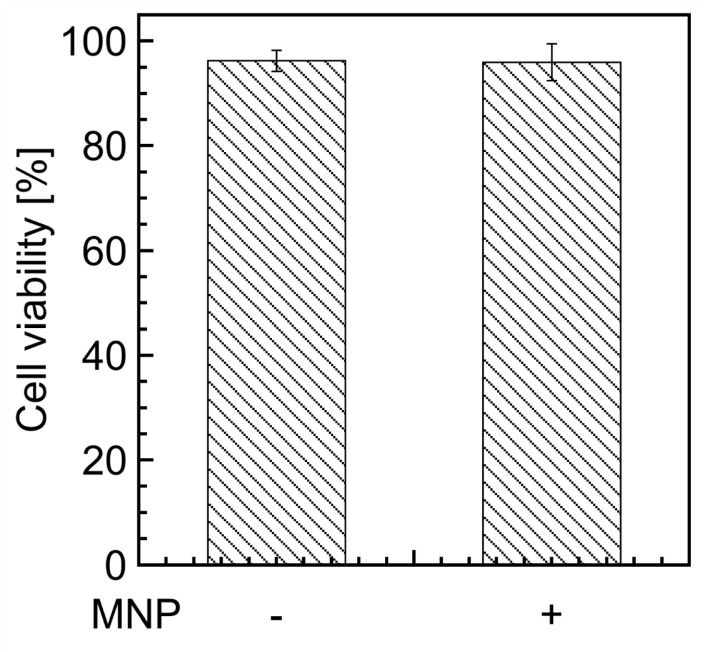


**Figure S3**. Viability of HeLa cells in adding PEI-coated MNPs. HeLa cells were seeded in 35-mm dishes at a density of 100,000 cells/well. One day after the incubation, PEI-coated MNPs of 2 μg/mL were added to each dish. The method of incubation and preparation of MNP was the same as that for the cell manipulation experiment. Two days after incubation, cell viability was evaluated by trypan blue dye exclusion test using an automated cell counter (AMQAF2000; Thermo Fisher Scientific Inc.).


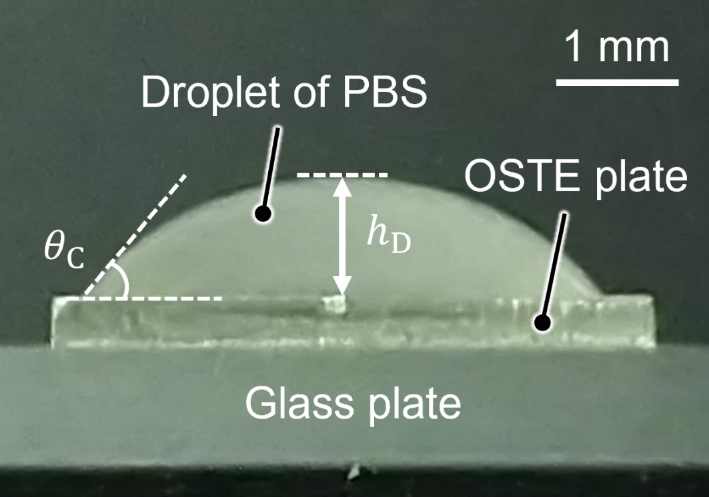


**Figure S4.** Photograph of the PBS droplet on the OSTE plate. *θ*_C_ is the contact angle between the basal plane of the droplet and the plate. *h*_D_ is the height of the droplet.


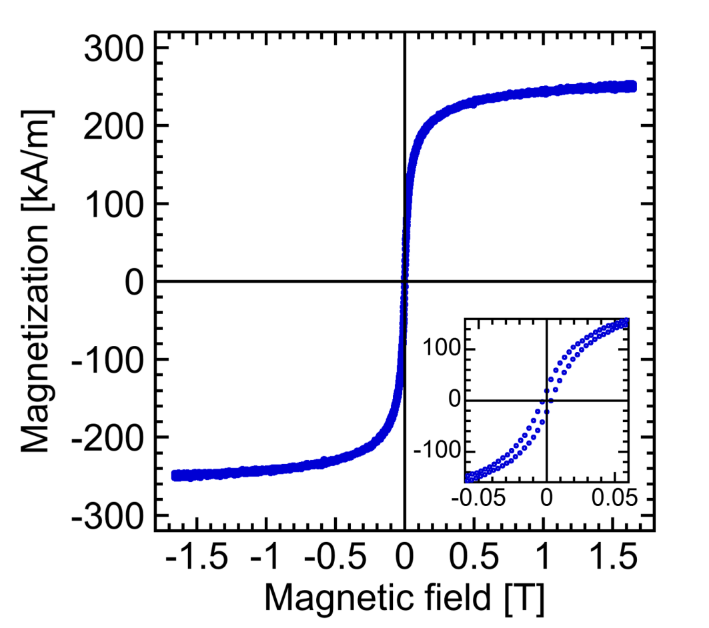


**Figure S5.** Magnetization curve of PEI-coated MNPs solidified by epoxy resin under a direct current magnetic field. Inset shows the magnified graph around the original point.


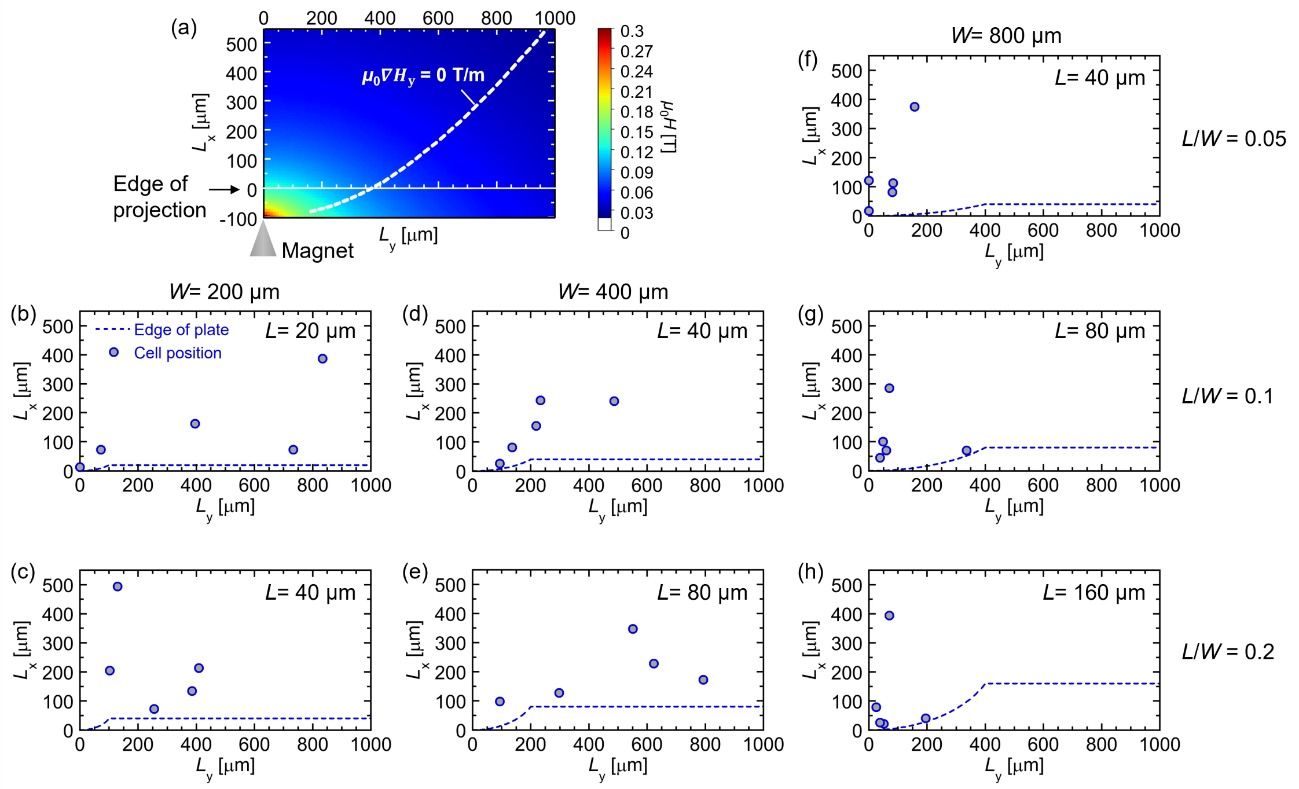


**Figure S6.** (a) Distribution of the magnetic field strength *μ*_0_*H* in a cell position from the edge of the projection in *x*- and *y*-axis, *L*_x_ and *L*_y_, respectively. At the dotted curve, *μ*_0_$\nabla$*H*= 0 T/m. The position of manipulated cells and the plate are respectively shown by plots and dotted curves in (b) *W*= 200 μm and *L*= 20 μm, (c) *W*= 200 μm and *L*= 40 μm, (d) *W*= 400 μm and *L*= 40 μm, (e) *W*= 400 μm and *L*= 80 μm, (f) *W*= 800 μm and *L*= 40 μm, (g) *W*= 800 μm and *L*= 80 μm, and (h) *W*= 800 μm and *L*= 160 μm.


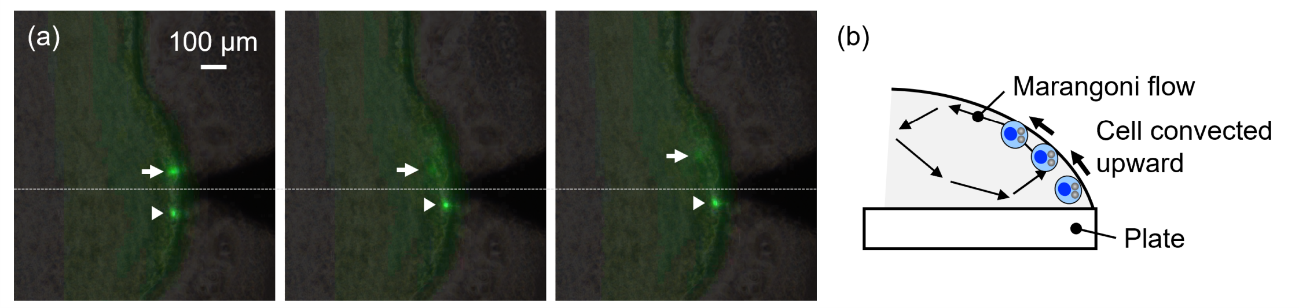


**Figure S7.** (a) Phase contrast images of the plate overlapped with fluorescent images of a cell manipulated on the plate at *W*=800 μm and *H*=160 μm. The cell indicated by white arrowheads approached the front edge of the projection under magnetic force. The cell indicated by white arrows moved upward and flowed off. (b) Schematic depicting the cell moving upward and flowing off under the effect of the Marangoni flow.
